# A Pouch, a Ridge, and a Thrombus

**DOI:** 10.1016/j.case.2026.05.009

**Published:** 2026-06-30

**Authors:** Satya Preetham Gunta, Thomas Schultz, Daniel Read, Andrew Blanker, Laura Beacher

**Affiliations:** a Division of Cardiovascular Medicine, Department of Internal Medicine, Medical College of Wisconsin, Milwaukee, Wisconsin; b Medical College of Wisconsin, Milwaukee, Wisconsin

**Keywords:** Left atrial septal pouch, Left atrial septal ridge, Thrombus, Atrial fibrillation

## Abstract

• LASP and LASR are common structural atrial findings of debated clinical significance.
• LASP and LASR may be associated with thrombus formation and thromboembolic events.
• Identification of atrial septal anomalies requires multimodal imaging techniques.
• LASP-associated thrombus in those with AF poses additional clinical challenges.

## Graphical abstract

**Figure:**
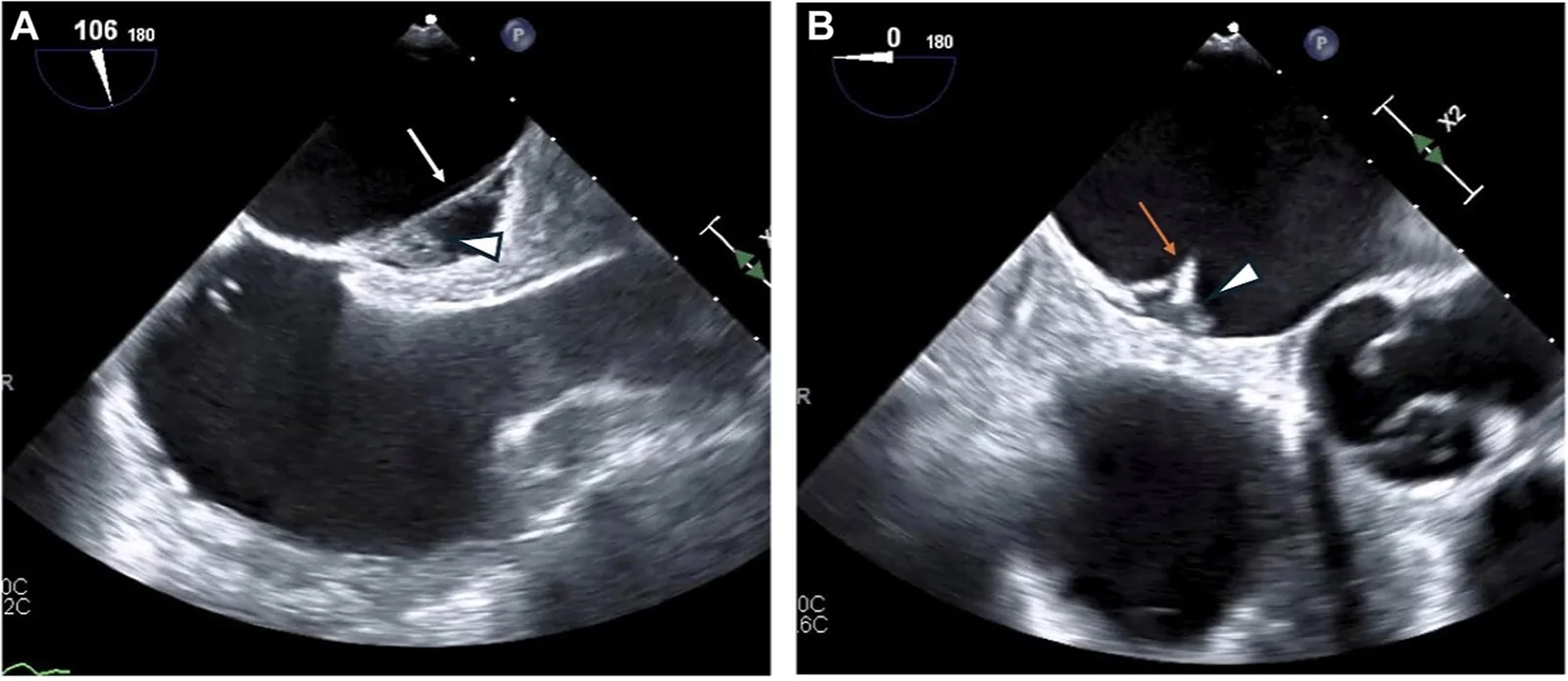

## Introduction

A left atrial septal pouch (LASP) is a pocket-like cavity formed on the left atrial side of the interatrial septum (IAS) due to incomplete fusion of the septum primum and septum secundum. While the LASP may create a region of low flow and serve as a nidus for thrombus formation, its clinical significance remains debated. A left atrial septal ridge (LASR) refers to localized thickening of the IAS, typically in the region of the fossa ovalis. Its presence can complicate transseptal procedures by altering septal morphology and necessitating careful preprocedural planning. Recognition of these entities can be challenging due to limited awareness, poor visualization of the left atrium (LA) on transthoracic echocardiography (TTE), and the need for advanced imaging modalities such as three-dimensional (3D) transesophageal echocardiography (TEE) or cardiac computed tomography (CCT). We present a unique case of coexistent LASP and LASR associated with a thromboembolic event, highlighting diagnostic challenges and clinical implications.

## Case Presentation

A 78-year-old man with a history of recurrent subarachnoid hemorrhage (SAH), hypertension, and newly diagnosed atrial fibrillation (AF) not on an oral anticoagulant underwent a TTE for workup of AF. The TTE demonstrated normal biventricular systolic function and left atrial enlargement. A pedunculated, mobile echogenic mass measuring 11 × 16 mm (Figure 1, Video 1) was attached to the IAS, raising suspicion for a left atrial myxoma. Following an in-depth discussion with the patient regarding the imaging findings, the patient elected to leave and return for an outpatient TEE, which was ordered for further assessment.

**Figure 1:**
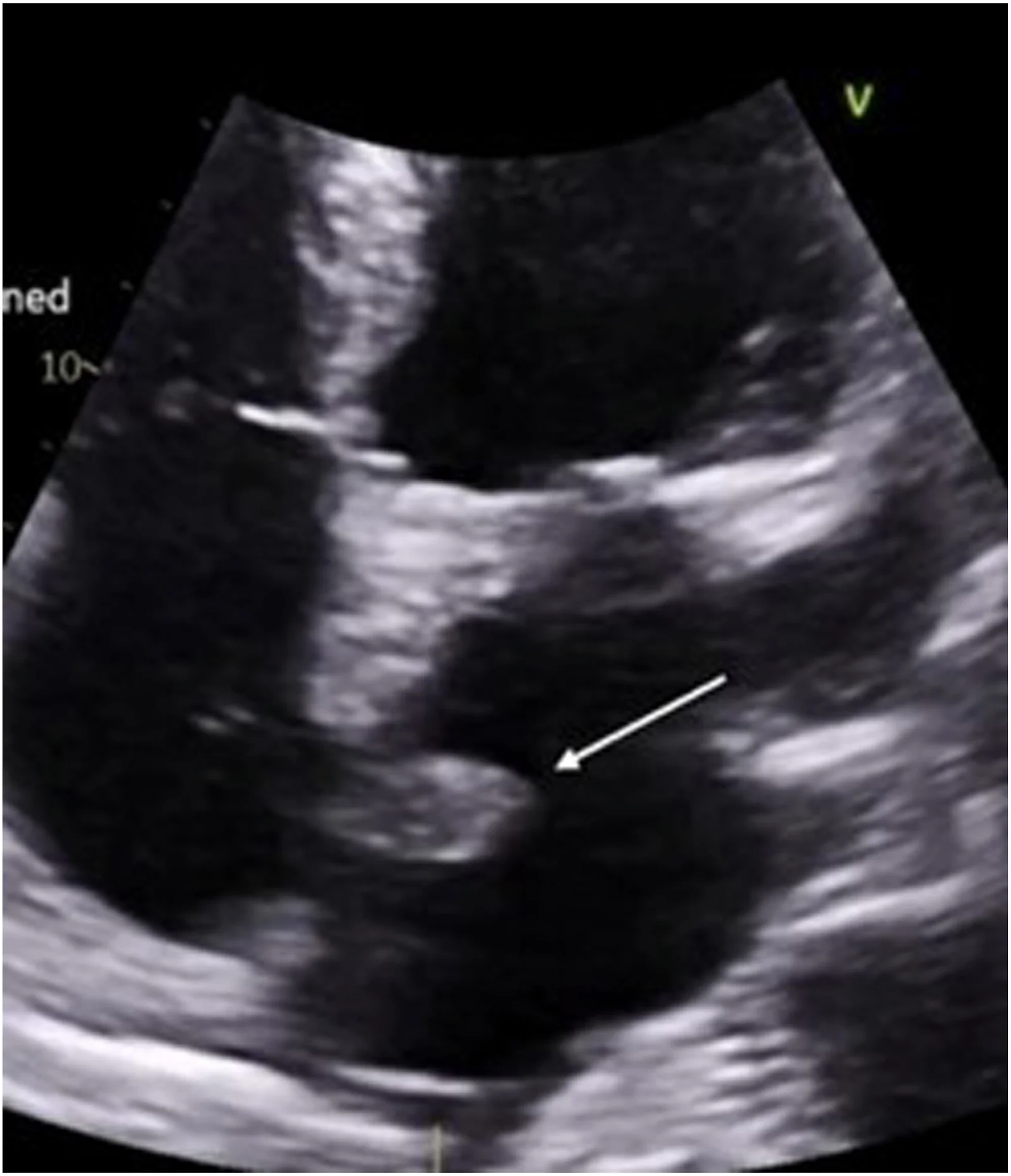
Two-dimensional TTE, apical 4-chamber focused systolic view, demonstrates a pedunculated, left atrial mass *(arrow)* attached to the septum, resembling a myxoma.

When the patient returned 11 days later for the TEE, they reported acute onset left thigh pain that had begun several days after the TTE. The TEE ruled out a left atrial appendage (LAA) thrombus. The pedunculated mass was no longer visualized. A linear “stalk-like” structure was visualized arising from the IAS and extending into the LA (Figure 2, Video 2). There was a small mass at its base, adherent to the septum (Figure 2A, arrowhead); 3D imaging was not performed at that time.

**Figure 2:**
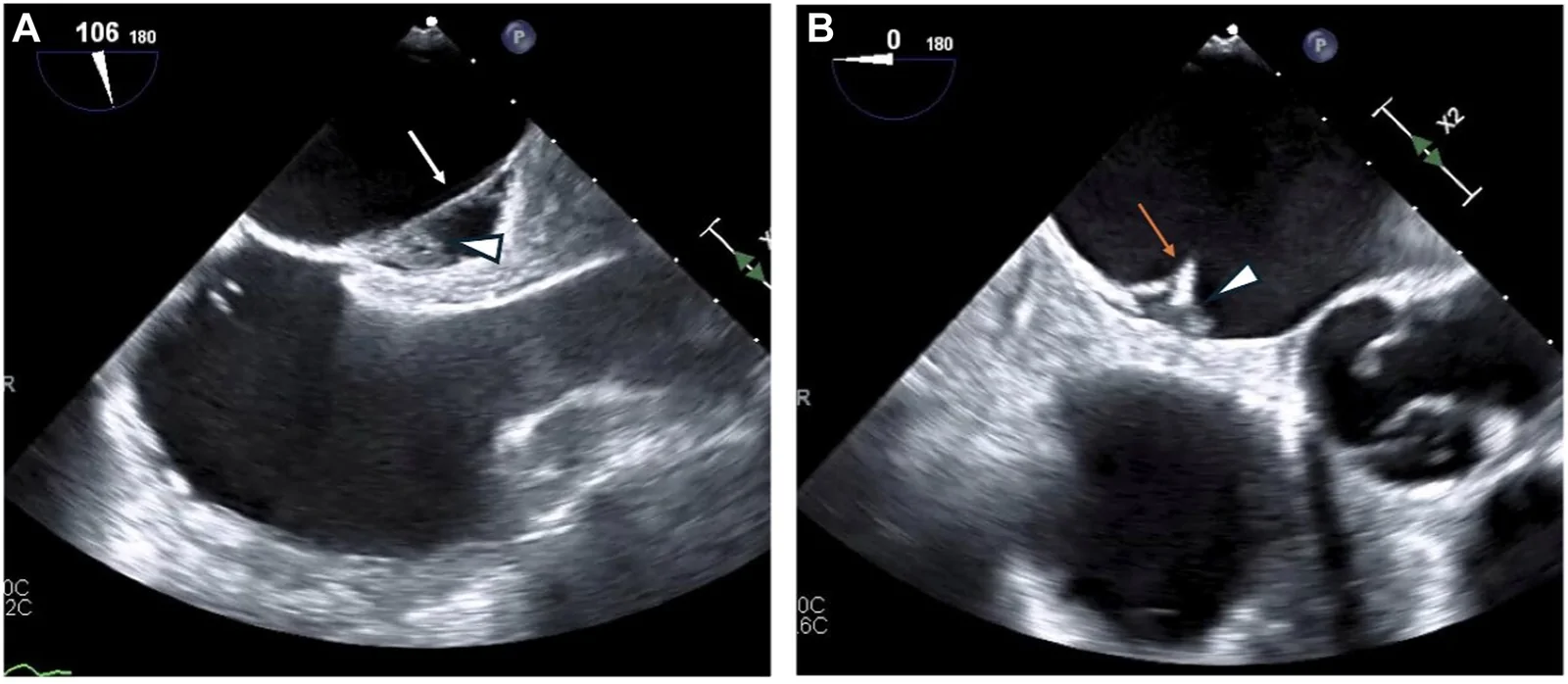
Two-dimensional TEE, midesophageal bicaval (106°) **(A)** and short-axis (0°) **(B)** views, demonstrates the LASP *(white arrow)* containing the thrombus-like, echogenic mass *(arrowhead)* and thickening of the IAS in the region of the fossa ovalis, creating a stalk-like appearance consistent with the LASR *(orange arrow)*.

Following the procedure, the patient continued to have worsening thigh pain. Given that the left atrial mass seen on TTE was no longer visualized on TEE, systemic embolization was suspected, and computed tomography (CT) angiography of the chest, abdomen, pelvis, and legs was obtained. The CT angiogram revealed complete occlusion of the mid-left superficial femoral artery with collateral reconstitution and a partial occlusion of the profunda femoris (Figure 3). The patient underwent successful thromboembolectomy, and histopathology ruled out myxoma and showed thrombotic material. The patient's pain resolved postprocedure, and they were discharged on a direct oral anticoagulant with close monitoring given the history of recurrent SAH. A repeat TEE was scheduled to evaluate for an interatrial shunt and possible paradoxical embolism.

**Figure 3:**
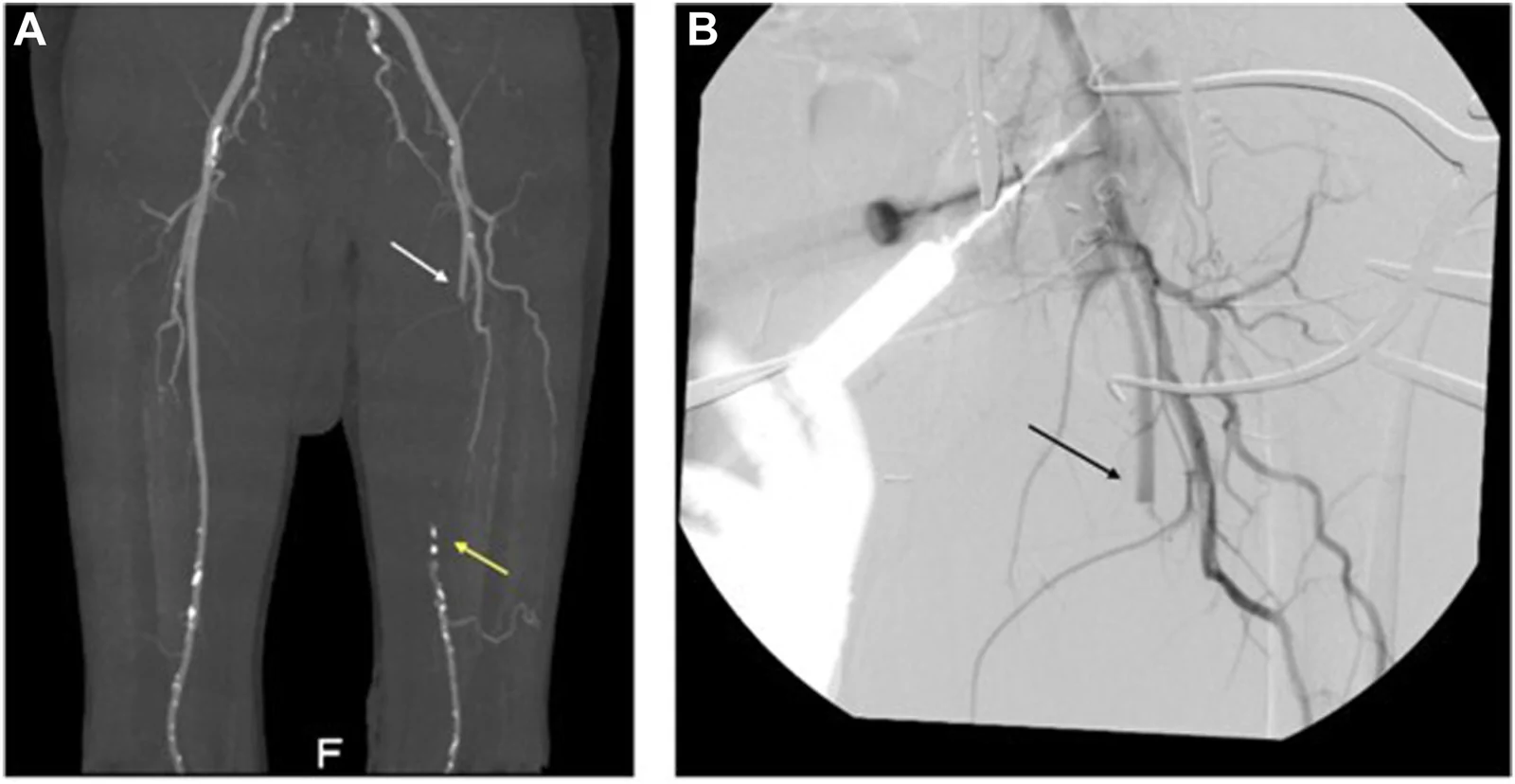
Computed tomography angiography, dissection protocol with reconstruction of the aortoiliac system **(A)** and invasive angiography of the left femoral artery **(B)**, demonstrates abrupt occlusion of the mid- left superficial femoral artery (SFA; *white and black arrows*) for approximately 19 cm with reconstitution of the distal left SFA *(yellow arrow)* and popliteal artery.

Retrospective review of the initial TTE (Figure 4, Video 3), TEE, and CT angiography (Figure 5) of the chest imaging showed the possibility that the “stalk-like” structure was the incompletely visualized tissue membrane extending from the septum across the LA.

**Figure 4:**
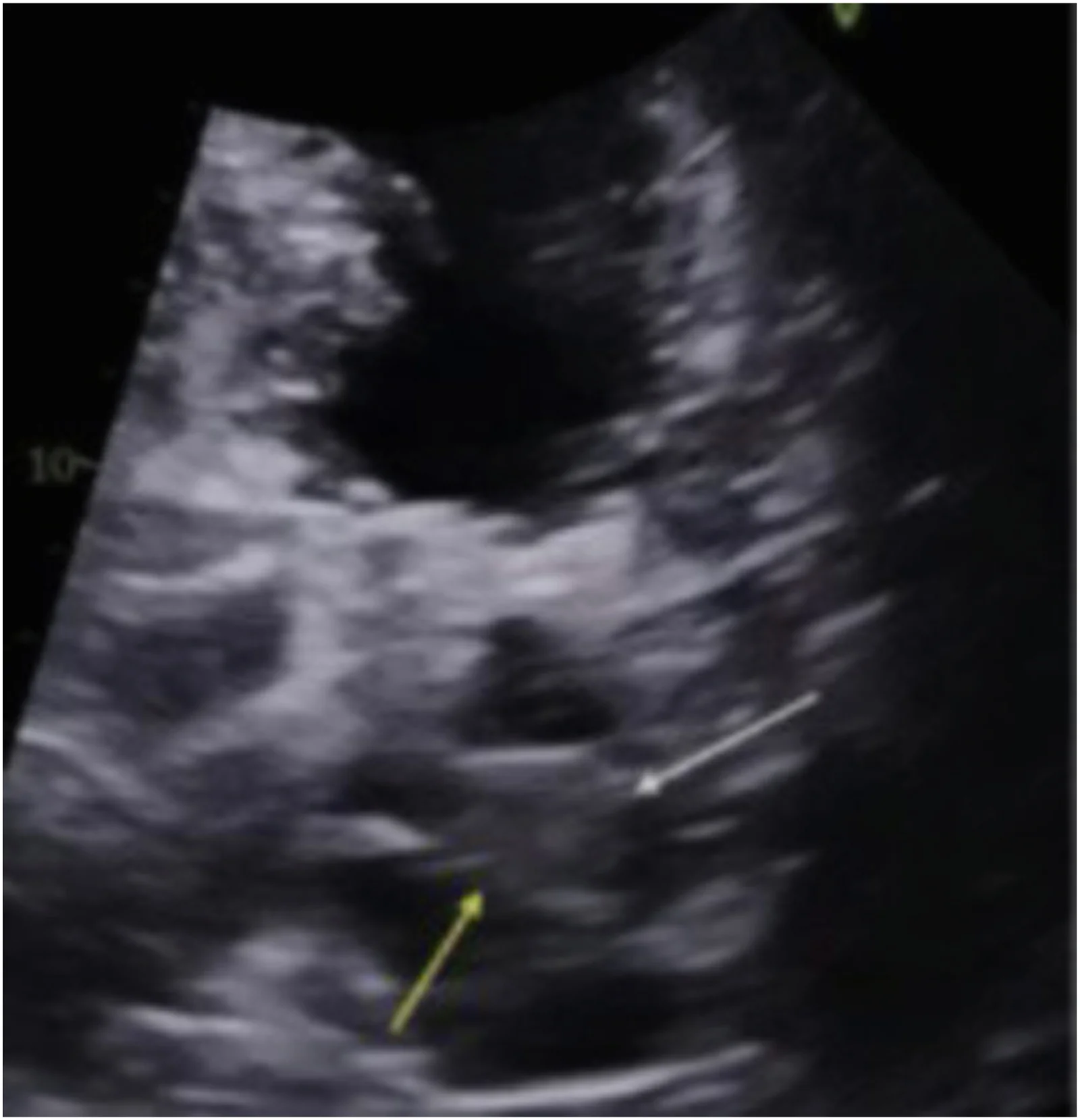
Two-dimensional TTE, apical-2-chamber systolic view, demonstrates the left atrial mass *(white arrow)* and a possible membrane *(yellow arrow)* extending anteroposteriorly in the LA.

**Figure 5:**
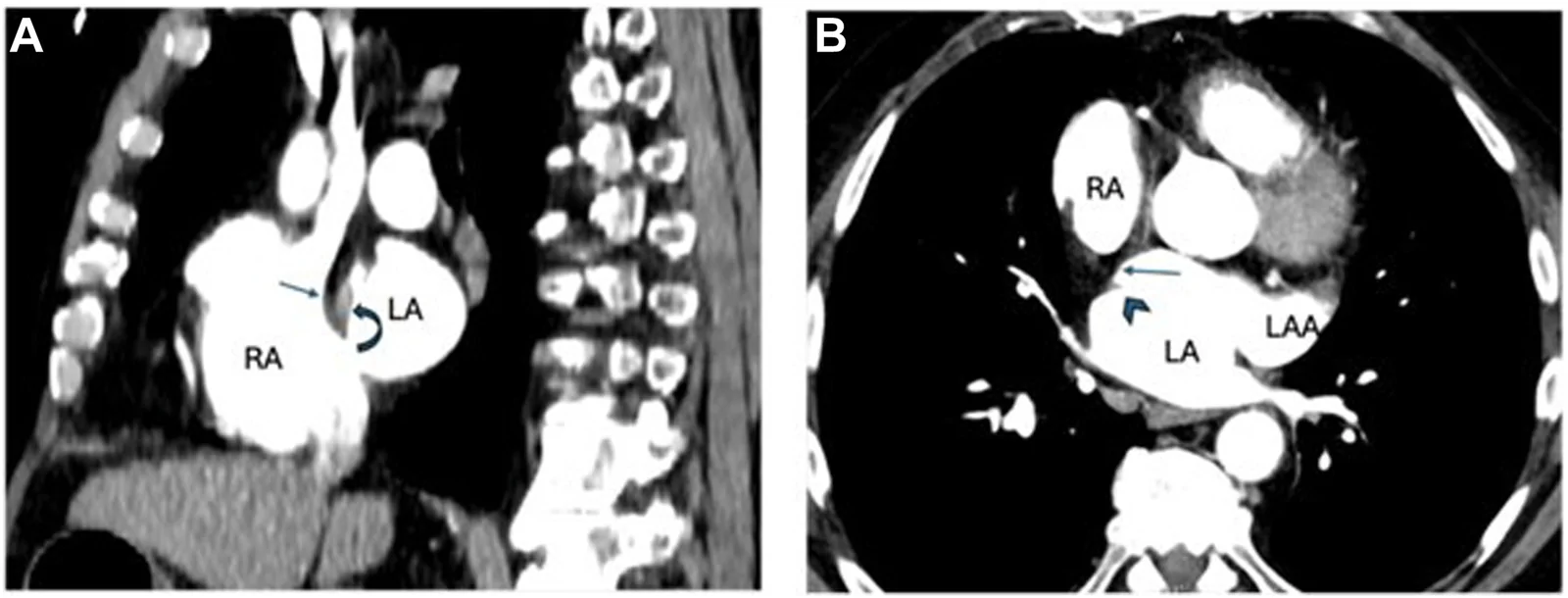
Computed tomography angiography, dissection protocol, sagittal **(A)** and axial **(B)** views, demonstrates the IAS *(straight arrows)*, the LASP with contained hypodense material within it *(curved arrow)* suggestive of thrombus, and the LASR *(chevron)*. *RA*, Right atrium.

On follow-up 3D TEE (Figure 6, Videos 4 and 5), detailed multiplanar reconstruction demonstrated an LASP bounded by the septum primum, posterior roof of the LA, and an LASR forming the free wall. There was no residual thrombus. A bubble study and color-flow Doppler imaging excluded an atrial-level shunt. The patient remained in persistent AF but had no thrombus in the LAA.

**Figure 6:**
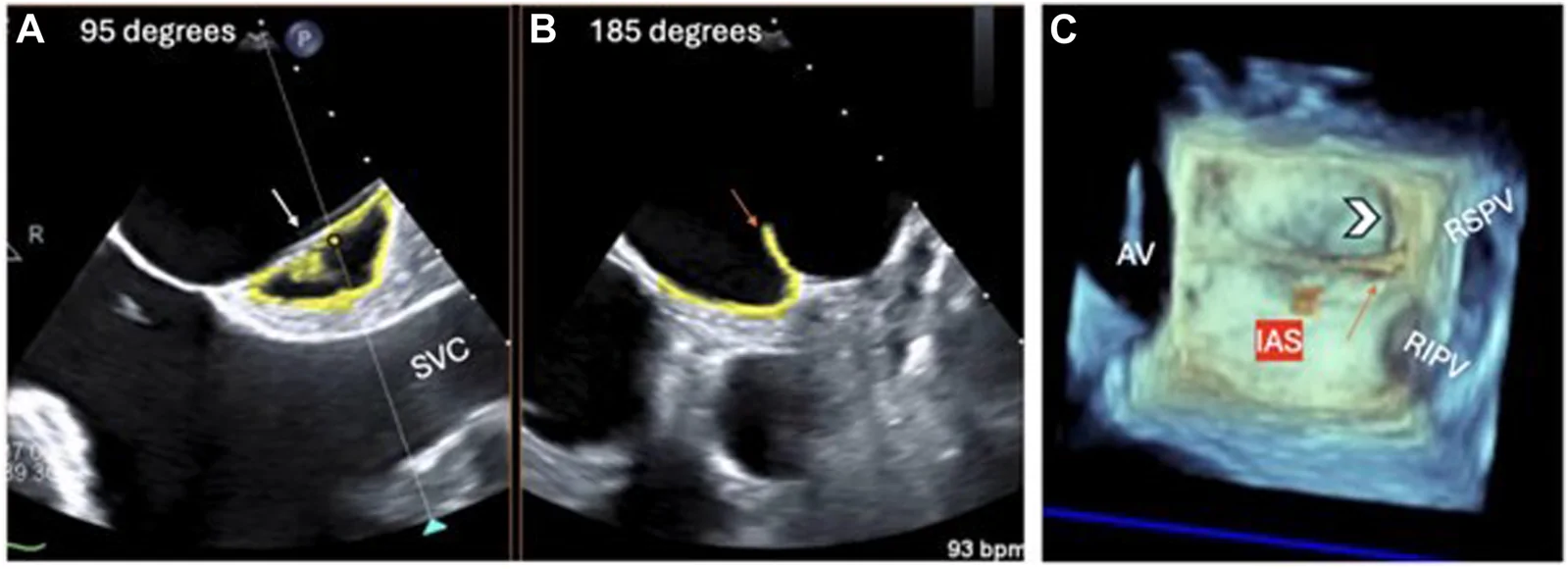
Three-dimensional TEE, midesophageal, simultaneous orthogonal, 2D long-axis **(A)** and short-axis **(B)** views and volume-rendered short-axis reconstruction from the atrial perspective **(C)** obtained 11 days after index presentation, demonstrates the LASP *(yellow highlight)* without contained echogenic mass, the LASR *(orange arrows)*, and the IAS and LASP *(chevron)*. *AV*, Aortic valve; *RIPV*, right inferior pulmonary vein; *RSPV*, right superior pulmonary vein; *SVC*, superior vena cava.

It was concluded that thrombus formation occurred within the LASP, likely due to low flow associated with AF, and subsequently embolized to the femoral artery. The echo dense mass initially mistaken for a myxoma on TTE represented this thrombus. The stalk-like structure on the initial TEE was the incompletely visualized LASR, not a stalk or a membrane. Given the history of recurrent SAH, LAA occlusion had been considered for stroke prevention. However, because LASP-related thromboembolic risk would persist despite LAA closure, the patient was no longer a candidate for device occlusion.

## Discussion

Left atrial septal pouch arises from the incomplete fusion of the septum primum and septum secundum during embryologic development. Complete fusion produces a normal septum, while absent fusion results in a patent foramen ovale. Partial fusion creates a pouch-like cavity, which can open into either the right atrium (right atrial septal pouch) or, more commonly, the LA (Figure 7). In autopsy studies, LASP is observed in up to 40% of specimens, while right atrial pouches occur in about 4%.^1^ The LASR represents localized thickening of the IAS, often in the fossa ovalis region, and may coexist with LASP, as in this case.

**Figure 7:**
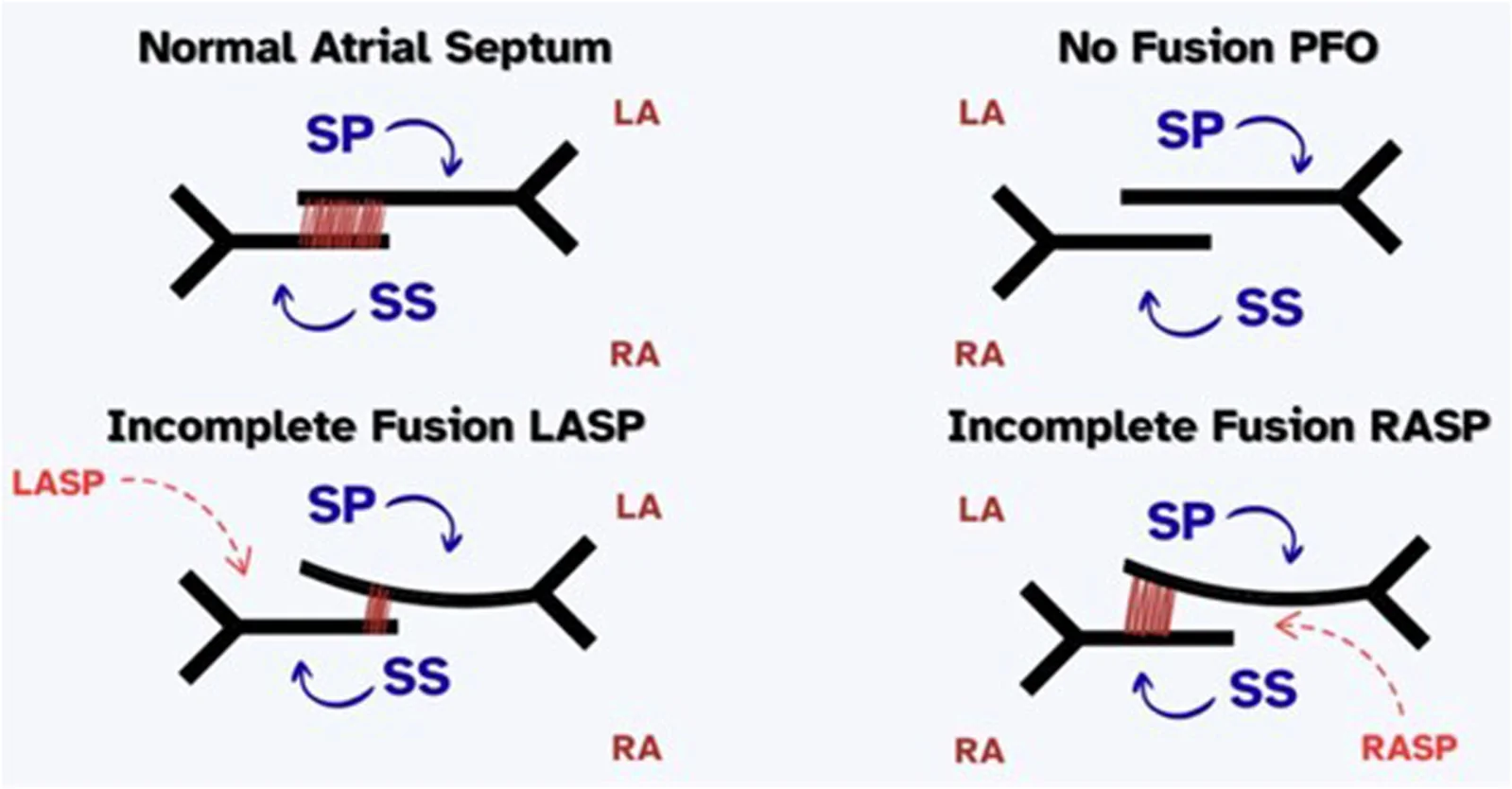
Schematic diagram depicting the embryology of left and right atrial septal pouches. *PFO*, Patent foramen ovale; *RA*, right atrium; *RASP*, right atrial septal pouch; *SP*, septum primum; *SS*, septum secundum.

Accurate identification of LASP and LASR relies on advanced and often multimodal imaging. Transthoracic echocardiography, frequently the first-line imaging study, may reveal large thrombi within an LASP, potentially mimicking a left atrial myxoma as in this case.^2^ Transesophageal echocardiography, especially in the bicaval view, is the most sensitive modality for visualizing LASP.^3^ Biplane and 3D imaging, additionally, are essential for delineating the spatial relationship between the pouch, ridge, and surrounding structures. In this case, 3D TEE clarified the diagnosis and ruled out cor triatriatum or artifact. Plane cropping, two-dimensional gain, multibeat acquisition, and real-time multiplanar reconstruction may additionally help with image optimization and demonstration.

When evaluating atrial structural abnormalities and masses, nonechocardiographic imaging including CCT and cardiovascular magnetic resonance (CMR) may offer supplemental information through enhanced tissue characterization and contrast resolution. Left atrial septal pouch and LASR can be identified on CCT, although their characteristically subtle morphology may necessitate multiplanar reconstruction for adequate visualization. Complications such as left atrial thrombi are readily apparent on CCT without supplemental reconstruction. Conventional steady-state free precession sequences on CMR offer limited evaluation of the IAS. When a left atrial mass is identified, CMR can offer a distinct advantage through superior tissue characterization. In particular, the long inversion time (long TI) sequence demonstrates a diagnostic accuracy of 99.2% for left atrial and LAA thrombus.^4^

The thromboembolic risk associated with LASP remains uncertain.^5^, ^6^, ^7^ While incidental LASP generally does not alter management, the presence of visualized thrombus warrants anticoagulation. Left atrial septal pouch and LASR also have procedural relevance, complicating transseptal access and emphasizing the need for pre- and intraprocedural 3D imaging. Evidence suggests that coexistent AF or other thrombogenic factors may amplify stroke risk in LASP patients.^8^, ^9^, ^10^ With the growing use of LAA occlusion devices and catheter ablation, the residual risk of thromboembolism from LASP in such patients remains unexplored and merits further study.

## Conclusion

This case illustrates coexisting LASP and LASR complicated by thrombus formation and systemic embolism, establishing a plausible causal link between LASP and thromboembolic events. Advanced multimodality imaging, particularly 3D TEE, plays a central role in identifying these anatomically complex and clinically significant entities that may otherwise be missed or misidentified.

## Ethics Statement

The authors declare that the work described has been carried out in accordance with The Code of Ethics of the World Medical Association (Declaration of Helsinki) for experiments involving humans.

## Consent Statement

Complete written informed consent was obtained from the patient (or appropriate parent, guardian, or power of attorney) for the publication of this study and accompanying images.

## Funding Statement

The authors declare that this report did not receive any specific grant from funding agencies in the public, commercial, or not-for-profit sectors.

## Disclosure Statement

The authors reported no actual or potential conflicts of interest relative to this document.

## Supplementary Data

Video 1 Two-dimensional TTE, apical 4-chamber focused view, demonstrates a pedunculated, mobile left atrial mass attached to the septum, resembling a myxoma.

Video 2 Two-dimensional TEE, midesophageal bicaval (106 degree) view, demonstrates the LASP containing the thrombus-like echogenic mass within and a mobile, filamentous structure in the right atrium with a prominent Eustachian valve.

Video 3 Two-dimensional TTE, zoomed apical 2-chamber view, demonstrates a pedunculated, mobile left atrial mass resembling a myxoma and a membrane-like structure extending anteroposteriorly in the LA.

Video 4 Three-dimensional TEE, midesophageal, simultaneous orthogonal, two-dimensional long-axis (A) and short-axis (B) views obtained 11 days after the index presentation, demonstrates the LASP and LASR without the previously noted echogenic mass.

Video 5 Three-dimensional TEE, midesophageal, volume-rendered short-axis reconstruction from the atrial perspective obtained 11 days after index presentation, demonstrates the LASP without contained echogenic mass and the LASR.

## References

[1] Krishnan S.C., Salazar M. (2010). Septal pouch in the left atrium: a new anatomical entity with potential for embolic complications. JACC Cardiovasc Interv.

[2] Shimamoto K., Ishii K., Takahashi T., Inoue I. (2014). Thrombus in the left atrial septal pouch mimicking myxoma. J Clin Ultrasound.

[3] Zisa D., Pescetelli I., Cerietti E., Halin N.J., Reddy P., Patel A.R. (2020). Ridges and pouches: a case series of anomalous atrial septal fusion. CASE (Phila).

[4] Kitkungvan D., Nabi F., Ghosn M.G., Dave A.S., Quinones M., Zoghbi W.A. (2016). Detection of LA and LAA thrombus by CMR in patients referred for pulmonary vein isolation. JACC Cardiovasc Imaging.

[5] Tugcu A., Yildiz M., Ozeke O., Rundek T., Homma S., Sacco R.L. (2010). Septal pouch in the left atrium and risk of ischemic stroke. JACC Cardiovasc Imaging.

[6] Sun J.P., Xu T.Y., Li L., Lee A.P., Chen M., Zhang B. (2016). Prevalence of atrial septal pouch and risk of ischemic stroke. Int J Cardiol.

[7] Holda M.K., Koziej M. (2018). Left-sided atrial septal pouch as a risk factor of cryptogenic stroke: a systematic review and meta-analysis. Cerebrovasc Dis.

[8] Buchholz S., Schlüter M., Kriegsmann J., O'Rourke M., Feneley M.P. (2012). Thromboembolic stroke with concurrent left atrial appendage and left atrial septal pouch thrombus. Int J Cardiol.

[9] Strachinaru M., van Rosendael P.J., van der Kley F., Gazagnes M.D. (2017). The left atrial septal pouch as a risk factor for stroke: a systematic review. Arch Cardiovasc Dis.

[10] Strachinaru M., van Rosendael P.J., van der Kley F., Morissens M., Costescu I., Catez E. (2017). The left atrial septal pouch as a possible location for thrombus formation. J Cardiovasc Med (Hagerstown).

